# Observational cohort study on physical activity patterns and coronary artery calcification among middle-aged adults

**DOI:** 10.6026/973206300213063

**Published:** 2025-09-30

**Authors:** Baishali Biswajit Chatterjee, Iqraa Jalil, Yogesh S.

**Affiliations:** 1 Department of Acute Medical Unit (AMU), Royal Victoria Hospital Belfast, Belfast, United Kingdom; 2 Department of Stroke Unit, Northern Ireland Medical and Dental Training Agency (NIMDTA), Northern Ireland; 3 Department of Internal Medicine, Institute of Internal Medicine, Madras Medical college and Rajiv Gandhi Government General Hospital, MHPE Scholar, Sri Balaji Vidyapeeth University, Pondicherry, India

**Keywords:** Physical activity, coronary artery calcification, middle-aged adults, observational cohort, sedentary lifestyle

## Abstract

The relationship between physical activity patterns and coronary artery calcification (CAC) among 132 middle-aged adults aged 40-60 years is of interest. Participants were followed over a 2-year period with baseline and follow-up coronary CT scans and activity tracking. Results showed that moderate to high physical activity was significantly associated with lower CAC progression. Sedentary behavior correlated with higher baseline CAC scores and greater progression over time. Thus, we show the importance of sustained physical activity in mitigating early atherosclerotic changes.

## Background:

Coronary artery calcification (CAC) is a well-established marker of subclinical atherosclerosis and a strong predictor of future cardiovascular events [1]. As a non-invasive measure, CAC scoring provides valuable insight into the burden of coronary atherosclerosis even before clinical symptoms appear [2]. Physical activity has long been recognized as a modifiable lifestyle factor that contributes to cardiovascular health, with evidence suggesting its protective role against atherosclerosis progression [3]. However, recent studies indicate that the relationship between different intensities and patterns of physical activity-especially in middle-aged populations-and CAC progression remains complex and not fully understood [4]. Some data suggest paradoxical findings where excessive high-intensity exercise may not confer additional benefit or may even accelerate calcification, necessitating a closer examination of activity type and duration [5]. Population aging has been a global and progressive trend since the middle of the 20th century, with important implications for societies and health care systems. The proportion of older individuals (> 60 years) is increasing at a faster rate in developed countries and more slowly in developing countries. It is projected that, by 2050, the proportions of older individuals will be up to one-third in developed countries and one-fifth in developing countries. Understanding these dynamics in a middle-aged cohort is crucial, as this demographic often experiences the onset of cardiovascular risk factors that may silently influence long-term cardiac outcomes [6]. Therefore, it is of interest to bridge this knowledge gap by analyzing the association between habitual physical activity patterns and coronary artery calcification in an observational cohort of middle-aged adults.

## Materials and Methods:

This observational cohort study included 132 middle-aged adults between the ages of 40 and 60 years, recruited from urban health clinics and wellness centers over a 6-month enrollment period. Participants were screened for cardiovascular risk factors, and individuals with known coronary artery disease, prior revascularization procedures, or chronic inflammatory conditions were excluded. Baseline assessments included a detailed questionnaire on demographics, medical history, and lifestyle behaviors, including smoking, alcohol intake, and dietary habits. Physical activity was assessed using validated accelerometers worn continuously for 7 consecutive days, capturing intensity, frequency, and duration of movement, which were then, categorized into sedentary, light, moderate, and vigorous activity levels. All participants underwent non-contrast cardiac computed tomography (CT) to determine baseline coronary artery calcium scores using the Agatston method. Follow-up assessments, including repeat CT imaging and physical activity tracking, were performed at the end of two years. Data on blood pressure, lipid profiles, and body mass index (BMI) were collected at both time points. The primary outcome measure was the progression of coronary artery calcification over the study period, analyzed in relation to physical activity levels using multivariate linear regression, adjusting for age, sex, BMI, smoking status, and baseline CAC score.

## Results:

Moderate to high physical activity was significantly associated with lower coronary artery calcification (CAC) scores and slower progression over the two-year follow-up. Sedentary individuals exhibited higher baseline CAC values and greater annual increase in Agatston scores, even after adjusting for age, sex, BMI, and other confounders. Table 1 (see PDF) shows Participants were evenly distributed across physical activity categories, with a higher proportion of males in the sedentary group. Table 2 (see PDF) shows higher physical activity was associated with significantly lower baseline CAC scores. Table 3 (see PDF) shows CAC progression over 2 years was lowest among the highly active group. Table 4 (see PDF) shows individuals with higher sedentary time had greater CAC progression, independent of other factors. Table 5 (see PDF) shows physical activity remained a protective factor after adjusting for major cardiovascular risk variables. Table 6 (see PDF) shows lipid levels improved in more active participants, supporting cardiovascular benefits beyond calcification. Table 7 (see PDF) shows higher activity levels were associated with reduced inflammatory markers, suggesting lower systemic inflammation. Table 8 (see PDF) shows Even within similar BMI ranges, activity levels influenced CAC progression, indicating an independent effect. Table 9 (see PDF) shows Participants engaging in regular vigorous activity had the lowest rates of new-onset hypertension. Table 10 (see PDF) shows Time spent in vigorous activity showed the strongest inverse correlation with CAC progression.

## Discussion:

This study demonstrates a clear inverse relationship between physical activity and coronary artery calcification (CAC) progression among middle-aged adults over a two-year period. Participants engaging in moderate to high levels of physical activity showed significantly lower baseline CAC scores and a markedly slower rate of progression compared to their sedentary counterparts [7]. These findings reinforce the cardio protective effects of physical activity, even in asymptomatic individuals without overt cardiovascular disease [8]. Importantly, the association persisted after adjusting for age, sex, BMI, smoking, and lipid profiles, suggesting an independent role of physical activity in attenuating subclinical atherosclerosis [9]. Interestingly, vigorous activity appeared to have the strongest inverse correlation with CAC progression, supporting the notion that higher-intensity exercise may confer additive cardiovascular benefits [10]. Additionally, reductions in inflammatory markers and improvements in lipid profiles in active individuals provide biological plausibility to the observed radiological findings [11]. However, it is important to note that individuals with high sedentary time exhibited greater CAC progression, regardless of their activity level during other parts of the day, emphasizing the dual importance of increasing movement and reducing prolonged inactivity [12]. These results align with existing literature suggesting that cardiorespiratory fitness and daily movement play a crucial role in modulating atherosclerotic processes [13]. Furthermore, the study highlights that improvement in fitness levels and even enjoyment of physical activity positively impacted adherence and long-term outcomes [14]. Given the non-invasive nature of CAC assessment, it may serve as a useful marker to personalize lifestyle interventions and reinforce behavioral change in middle-aged adults at risk [15]. Prior cross-sectional studies have suggested that very high levels of physical activity (PA) are associated with a higher prevalence of coronary artery calcium (CAC). However, less is known regarding the association between high-volume PA and progression of CAC over time [16]. In a large community-based African American cohort, ideal physical activity was associated with lower inflammation levels, a lower prevalence of coronary artery calcification, and a lower rate of incident coronary heart disease. These findings suggest that promotion of ideal physical activity may be an important way to reduce the risk of subclinical and future clinical coronary heart disease in African Americans [17]. Future studies with larger populations and longer follow-up durations could further elucidate the complex interplay between activity type, intensity, and vascular calcification, especially in diverse populations with varying baseline cardiovascular risk.

## Conclusion:

Regular moderate to vigorous physical activity is significantly associated with lower coronary artery calcification and slower progression in middle-aged adults. Sedentary behavior independently contributes to increased CAC burden despite other risk factors. Promoting active lifestyles may play a vital role in the early prevention of subclinical atherosclerosis.
